# Observations of Oral Conditions in Their Relation to Body Functions*Extract from a paper published in the *Dental Digest* for May.

**Published:** 1917-07

**Authors:** Waite A. Cotton

**Affiliations:** New York City


### OBSERVATIONS OF ORAL CONDITIONS IN THEIR RELATION TO BODY FUNCTIONS *

BY WAITE A. COTTON, D.D.S., NEW YORK CITY

Pathological conditions in the mouth are symptoms of deranged body functions, as distinguishable and understandable as temperature, respiration and blood-pressure.

Up to the present it has been believed that there is no way of telling why the teeth in one mouth are susceptible to decay and in another mouth are immune.

I propose to show that there is a way of determining the etiology of dental decay and calcareous deposits.

It is a matter of common knowledge among dentists that their patients are divided into classes, and that these classes are distinguishable by well-known phenomena. For instance, every dentist has a class of patients who return

---

* Extract from a paper published in the *Dental Digest* for May.

to him frequently to have the tartar removed. This condition exists apparently in spite of the most persistent use of such methods of dental prophylaxis as are capable of employment in the hands of the layman. Another class is composed of those who uniformly exhibit the phenomena of both primary and recurrent caries, no matter how careful and competent the mechanical work has been, and in spite of conscientious oral prophylaxis. The teeth of one class show decay at the gum margin. Another class manifests no tendency to decay around the gum but does show decay between the teeth or on the morsal surface.

There is, apparently, no special connection between these classes. An individual whose teeth accumulate tartar may seemingly have extreme resistance against decay. Another whose teeth show marked susceptibility to decay will have no tendency to the accumulation of tartar.

A further puzzling instance is in line with the recent extensive investigations and wide-spread interest in pyorrhea. The question has arisen in the minds of most of us as to whether generalizations regarding pyorrhea are not misleading. It is evident that the harmful influences of pyorrhea, both in the mouth and upon the general well-being of the patient, are widely varying, and that one class with extensive pyorrhea will show practically no decay, while another class will have a considerable amount. In some mouths pyorrhea is easily and rapidly alleviated. In other mouths, where it is apparently less active in its progress and extensive in its involvement, it responds to treatment very slowly. There are also some cases which can only be partially relieved and never cured.

These examples and observations might be multiplied. They have puzzled all of us for many years. In my own practice I simply knew that there were some mouths that I could not keep in condition—that there were other mouths in which my work remained indefinitely.

Speculation and research led me to no conclusions which were of practical value in the care of those mouths which

I could not maintain in a noncarious condition in spite of every effort. In a word, some individuals' teeth tend to decay and the teeth of other individuals do not tend to decay; some individuals' teeth tend to accumulate tartar, and the teeth of other individuals do not tend to accumulate tartar.

Dental literature afforded little help. The solution apparently lies outside the scope of what has been hitherto regarded as dental science which, like other specialties in the therapeutic field, has been developing within itself. Just as the specialist on diseases of the skin today is rapidly coming to depend upon forces outside of his local applications for the ultimate and permanent remedy of his patients' affliction, so it seemed to me that I must look beyond the literature of technical and mechanical dentistry for the solution and remedy.

The final light was one of personal experience. I had been one of the class which rapidly accumulated tartar. In spite of dental floss and toothbrush I was obliged to have my teeth attended to every month—a condition persisting over several years. I tried every means known to dentistry to relieve this condition. After a time I broke down from overwork and nervous strain, and was compelled to place myself in the hands of my physician who had been especially interested in chronic toxemias and functional derangements and had done considerable work along these lines. He dismissed me with explanations and instructions as to the maintaining of a normal, reactive and functionating body. This was three years ago. On several of my subsequent monthly inspections for accumulated tartar I was very much surprised to learn that practically no tartar had accumulated, and that my teeth were free from evidence of decay.

*Decided alterations in my oral reactions had apparently been effected by changes in my body function.*

I realized that here was a field of obvious cause and effect which has never been explored by detailed clinical observation within the realms of dentistry.

I began to collect material observations on my patients. At first these were limited. Reflection on observations already made, however, led to further problems, further observations and more detailed study and definite experimentation.

Reviewing the work that I have done up to the present time, I find that a considerable part of whatever value I have achieved is due to the fact that I have made my observations upon people, who, year in and year out, were in more or less constant touch with me—and whose individual changes I was able to follow and to interpret in a way that would have been impossible in a dispensary practice. With more detailed statistical material, and more complete laboratory equipment, I have made a sufficient number of observations and experiments and have reached sufficient consistent deductions and classifications to enable me to present the work as far as it has gone.

I am advancing—and in subsequent papers shall elaborate and support a hypothesis which carries the science of dentistry beyond the mechanical repair of the teeth and establishes fundamental relations between the conditions of the teeth themselves and the reactions of the body.

The inhibition of dental decay and the protection of the oral cavity is dependent upon the normal functioning power of the mucous membrane; this in its turn depends on the establishment and maintenance of normal physiological conditions in the body as a whole, as contrasted with local conditions in the mouth.

The dentist can not hope to attain the ultimate goal in the prevention of dental decay until he can correctly diagnose an imperfect functioning mucous membrane, and is cooperating with the general practitioner or specialist for the correction of the pathological conditions causing the deranged oral mucous membrane. Unless such steps are taken all efforts of the dentist will leave the causes of dental decay untouched, and his work will be confined to repairing its ravages, often a hopeless and losing battle. —*Dental Digest.*

